# Supplementary material for: Learning curve and surgical outcome of robotic assisted colorectal surgery with ERAS program
Source: Sci Rep. 2022 Nov 29;12:20566. doi: 10.1038/s41598-022-24665-w (PMC9709162; doi:10.1038/s41598-022-24665-w)
Supplement: Supplementary file 2 — Supplementary Information 2. [file 41598_2022_24665_MOESM2_ESM.zip › RAL Raw data-Ñ[▒K/134/OP N_ñw╝╨░O▒Kñσ.pdf]

W92 / W92

臺中榮民總醫院
手術室護理記錄

[redacted]

基本資料

- 室別： 開刀房-17室，手術科別： CRS，手術類別： 常規，日期： 2021/12/16
- 進入時間： 0730 ； 麻醉開始時間： 0750 ； Time Out時間： 0910 ； 劃刀時間： 0911 ； 手術結束時間： 1300 送出時間： 1330 ；
- 麻醉方式： GE
  診斷：Malignant tumor of sigmoid colon
  手術名稱：Robot Assisted Colectomy(L)
  手術局部用藥：NIL
  術前預防性抗生素：
  - 抗生素名稱一： Veterin 1GM IVD/由麻醉護理師給予
  - 時間： 0850
  - 上次簽章: 陳盈妤

  ，追加預防性抗生素：
- 手術醫師： 蔣鋒帆，陳瑢夫，陳威遠
- 麻醉醫師： VS 陳永銘
- 刷手護士A ： 歐真勤，時間： 0730-1100，刷手護士B ： 洪千淳，時間： 0730-1130，接替刷手一： 歐真勤，時間： 1130
  流動護士A ： 陳盈妤，時間： 0730，接替流動一： 洪千淳，時間： 1200

皮膚狀況

A-V Shunt： 無
術前： 完整
  記錄者： 上次簽章: 陳盈妤
術後： 完整
  記錄者： 上次簽章: 洪千淳

手術準備

手術臥式 單一手術臥式

-
  - 手術臥式： 截石臥式，NIL
  - 使用附件： 溫毯 40 ℃，頭圈，腳架，其它： 側身附件x4
  - 皮膚準備： 10%Aq Betadine，Hibiscrub，2% Chlorhexidine，其它： NIL
  - 電燒： 使用電燒: 電極片位置： 其它： 導電軟墊，記錄者： 上次簽章: 陳盈妤

止血帶T： 無

植入物

無

標本送檢

- 細菌培養 無
- 細胞學 無
- 冰凍切片 無
- 病理標本
  組織名稱一：S-colon
  ,
  組織名稱二：IMA root
  ,
  組織名稱三：Distal cut end
- 截肢 無

標本交付 標本： 3 袋，標本： 0 盒，標本： 0 瓶，細胞學： 0 管，Culture Tube： 0 管，被交付醫師： CR 陳瑢夫，

- 記錄者：

- 時間： 1230

傷口術後敷料

傷口敷料：　　　　　　　　　　　　　　傷口敷料： 無

- 請於圖上標示部位：

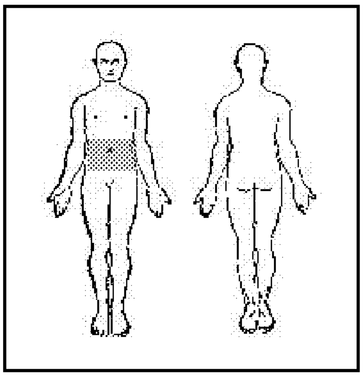

- 傷口敷料： Trocar:menpor+Gauze

導管

導管	天數	導管資訊	插入日期	插入時間	更換日期	拔除日期	拔除時間	拔除原因	預計拔除/更換	預計拔除/更換日期
Foley(1)	1	號碼:16 Fr.; 部位:膀胱;	2021/12/16	0815						
Epidural PCA(1)	1	部位:硬脊膜;	2021/12/16	0810						
Double Lumen CVC(1)	1	號碼:8Fr. ; 部位:; 象限:RU; 註記:fix 15cm;	2021/12/16	0805						
A-line (1)	1	號碼:20G ; 部位:左手;	2021/12/16	0820						

用物計數

- 手術中出血量： minimal c.c.
- 手術中尿量： 200 c.c.
-
    - 其它： 無
    - NIL

用物計數： 多切口同時縫合

- 1.器械： 正確，2.縫針： 正確，3.刀片： 正確，4.紗布： 正確，5.腹部墊： 未使用，6.紗球： 未使用，7.PEANUTS ： 未使用，8.紗條： 未使用，9.絲帶： 未使用，10.針頭： 未使用，11.Vessel loop： 未使用，12.Boots： 未使用，13.Jelco： 未使用，14.棉條： 未使用，
    - 第一次：
        - 刷手護士 ： 歐真勤
        - 流動護士： 上次簽章: 陳盈妤
        - 時間： 1130

    ,
    - 第二次：
        - 刷手護士 ： 歐真勤
        - 流動護士： 上次簽章: 陳盈妤
        - 時間： 1145

    ,
    - 第三次：
        - 刷手護士 ： 歐真勤
        - 流動護士： 上次簽章: 洪千淳
        - 時間： 1250

備註

備註： Davinci XI 30度:NO.2
達文西上機時間0945下機時間1130

- 記錄者： 上次簽章: 陳盈妤
- 時間： 0745

填寫護理人員 洪千淳

正式摘要

W92 / W92

doctorRank:B
